# Salivary Biomarkers (Opiorphin, Cortisol, Amylase, and IgA) Related to Age, Sex, and Stress Perception in a Prospective Cohort of Healthy Schoolchildren

**DOI:** 10.1155/2021/3639441

**Published:** 2021-11-12

**Authors:** Anna Krahel, Elzbieta Paszynska, Justyna Otulakowska-Skrzynska, Szymon Rzatowski, Amadeusz Hernik, Agnieszka Slopien, Tomasz Hanć, Paula Szczesniewska, Ewa Bryl, Yves Boucher, Marta Tyszkiewicz-Nwafor, Maria Gawriolek, Monika Dmitrzak-Weglarz

**Affiliations:** ^1^Department of Integrated Dentistry, Poznan University of Medical Sciences, Poznan 60-812, Poland; ^2^Department of Child and Adolescent Psychiatry, Poznan University of Medical Sciences, Poznan 60-572, Poland; ^3^Institute of Human Biology and Evolution, Faculty of Biology, Adam Mickiewicz University, Poznan 61-614, Poland; ^4^Laboratoire de Neurobiologie Orofaciale (EA 7543), UFR Odontologie, Université de Paris & Groupe Hospitalier Pitié Salpêtrière, 75006 Paris, France; ^5^Department of Psychiatric Genetics, Department of Psychiatry, Poznan University of Medical Sciences, Poznan 60-806, Poland

## Abstract

**Background:**

The use of easily accessible biomarkers for assessing young patients' health is weighty. This cohort study is aimed at measuring stress/immune biomarkers in the saliva of healthy school-age children and comparing subgroups according to age, sex, and stress perception. *Material and Methods*. 503 children under 12 years old (8.7 ± 1.3) were included with anthropometric evaluation (height, waist, hip circumference, body weight, and body mass index (BMI)). Levels of opiorphin (OPI), free cortisol, alpha-amylase (sAA), and secreted immunoglobulin (sIgA) were determined by quantitative assays (ELISA) in unstimulated saliva. Unpaired *t*-test, Welch test, and Mann–Whitney *U* test were applied for appropriate group comparisons, and the correlation between variables was analyzed with Spearman's rank coefficient. Results were considered significant at *p* < 0.05.

**Results:**

sIgA and sAA exhibited significant differences depending on age and sex: IgA (ng/mL): 86 ± 68.6 vs. 104.9 ± 72.1 for (6-7 y.o.) and (8-11 y.o.), respectively, and 108.1 ± 80.1 vs. 94.6 ± 62.2 for male and females, respectively; sAA (U/mL): 78.9 ± 54.4 vs. 100.5 ± 81.2 for (6-7 y.o.) and (8-11 y.o.). No difference related to age or sex between groups was observed for cortisol and OPI. However, OPI levels were higher and correlated to prior stress exposure in children (0.31 ± 0.4 vs. 0.26 ± 0.5 ng/mL, *p* = 0.031). sAA was negatively correlated to low mood self-declaration in children in the last two weeks (*r* = −0.10, *p* = 0.045).

**Conclusions:**

sIgA and sAA can be used as sex- and age-related biomarkers in children 6-12 y.o., which is not the case for free cortisol and opiorphin. However, OPI reflected previous exposure to stress, suggesting its use for evaluating stress-related changes in children.

## 1. Introduction

Psychological stress impairs homeostasis in many aspects, including immune dysregulation [1] with significant variability according to age and sex [2]. The use of easily accessible biomarkers is essential to assess these changes in fragile subjects, like children, especially in a context of exposure to stressful experiences during childhood, where invasive sampling procedures may increase psychological stress.

Saliva is a promising medium that has been used to evaluate both acute and chronic stresses [3]. Several salivary immune biomarkers are now considered to be sensitive and reliable readouts of mental stress in adult patients [4], primarily cortisol, alpha-amylase (sAA), secretory immunoglobulin IgA (sIgA), and opiorphin (OPI).

Cortisol has been often used to assess the activity of the hypothalamic-pituitary-adrenal (HPA) stress axis system and received extensive attention in research on stress physiology [5]. Life situations described as unpredictable or dangerous cause increased cortisol release. Cortisol secretion, which physiologically peaks at awakening and gradually decreases throughout the day, increases in acute stress situations before returning to normal levels after cessation of stress [6]. Long-term high levels of cortisol may exert harmful, deleterious effects on mood [7], obesity [8], and blood glucose levels [9]. Low levels have also been linked to pain, fatigue, high stress sensitivity, and stress-related disorders such as posttraumatic stress syndrome [10].

Salivary amylase (sAA) was also recently suggested as an indicator of sympathetic nervous system (SNS) activation but significantly different from cortisol. Typical sAA concentration reaches a peak in the late afternoon or evening [11]. In acute stress situations, sAA secretion may have additional distinct peaks [12]. sAA showed positive correlations with heart rate, pain intensity, and cortisol, suggesting HPA and SNS coordination [13].

IgA is a secreted glycoprotein, part of the adaptive immune system, that acts synergistically with other inherited mucosal defense factors, such as alpha-amylase, lactoferrin, and lysozyme [14]. Similarly, to cortisol, sIgA peaks in the morning and then gradually declines till the evening. Acute stress increases sIgA release, whereas chronic stress has an inhibitory effect, emphasizing the ability of the immune system to protect the body against disease [15].

Opiorphin (OPI) is a polypeptide recently discovered in human saliva, increasing the bioavailability of enkephalins acting on *μ*- and *δ*-opioid receptors in the central nervous system (CNS), therefore displaying analgesic and antidepressant properties [16–25]. Salivary OPI secretion depends on ovarian cycles and hormonal status [18], which are known to affect the trophism of oral tissues [26–29]. OPI also plays a role in oral pain [26, 28, 30] and mental stress [27].

These four biomarkers may be easily measured in saliva, but in variable amounts depending on age and sex, as well as in response to psychological stress [31]. The main objective of this exploratory study was therefore to compare salivary levels of opiorphin, cortisol, sAA, and sIgA using enzyme-linked immunosorbent assay (ELISA), in two groups of healthy children, 6-7 and 8-11 years old, a developmental age critical for mental disorder development [32]. Secondary objectives were to compare salivary biomarkers' levels in subgroups of subjects stratified by age, sex, anthropometric parameters, stress perception of prior distressing events, and mood.

## 2. Materials and Methods

### 2.1. Ethics

The study was approved by the Ethics Committee, Poznan University of Medical Sciences (resolution no. 542/14 from 6 December 2014). Parents or legal guardians gave written informed consent for the participation of their children in the study. Prior to the examination, the purpose of the study was explained to the children. They were also asked to express their opinions on the study.

### 2.2. Subjects

**Figure 1:**
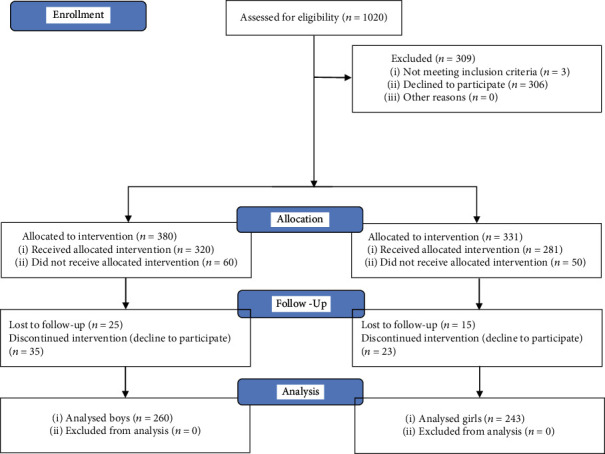
Study flowchart.

Subjects were healthy schoolchildren attending an urban district school in the years 2019-2020 (the northern, southern, eastern, and western parts of the Poznan agglomeration). Ten primary public schools were randomly selected in the aforementioned area and invited to participate in the study. Six schools agreed. During meetings with the parents, professionals explained the details of the study and its noninvasive nature. Then, parents registered their children for final participation. After a general interview, children were screened for inclusion/exclusion criteria. The final sample was then divided into two subgroups according to age: 6-7 (group 1) and 8-11 (group 2) years old (y.o.). The rationale for age categories was based on Poland's educational system and hormone-related changes in children at those ages. As puberty might affect opiorphin production, we excluded children over 12 y.o. since sex steroid impregnation usually does not occur before this age [33]. The scholar system in Poland includes 6 years of early education (6-11) followed by 4 years of primary school. Each cycle is related to different intellectual and social requirements and skills. We divided the early childhood education period into groups of 6 to 7- and 8 to 11-year-old children, to obtain homogenous groups regarding socio-cultural parameters. The study flowchart is illustrated in Figure 1.

### 2.3. Exclusion/Inclusion Criteria

Inclusion criteria were as follows (Table 1): children >6 and <12 years old, consent signed by parents, and study participation orally approved by the child.

Exclusion criteria were divided into four categories: (1) school attendance: child absent from school more than 4 weeks in the recent period; (2) general health: permanent somatic diseases, mental or neurodevelopmental disorders, hereditary disorders (first-degree relatives), pharmacotherapy, endocrine therapy, and dietary supplements; (3) oral health: urgent or nonroutine dental treatment, ongoing orthodontic treatment/wear of an orthodontic appliance, and any general or oral treatment prone to alter salivary flow and composition; (4) communication: inability to answer questions and lack of cooperation during physical measurements or salivary sampling.

### 2.4. Children Assessment: Mental Evaluation

MINI-KID, a proprietary structured questionnaire completed by a specialist in child and adolescent psychiatry [34–36], was used to exclude children presenting mental and neurodevelopmental disorders from the study. The MINI-KID questionnaire is a widely used screening tool designed to quickly identify children at risk of psychiatric disorders [36] with well-supported reliability and validity in its Polish version [37]. Finally, child stress experience in the past 6 months and assignment to the SE1 or SE0 subgroups were based on the answers given in the following part of the questionnaire: Traumatic Event Screening Inventory (TESI) (see Supplementary 2). Confirmation by any declaration of the stress experience caused inclusion or exclusion to subgroup SE1 (yes) or SE0 (no). The list of analyzed experiences was as follows:

“…Tick the events that took place in the child's life in the last 6 months”:

1. Life or health of the child was seriously endangered (yes/no)
2. Child witnessed an event in which the life or health of another person was at risk or someone died (yes/no)
3. Child experienced physical violence (beating, jerking, pushing, burning, choking, forcing to sexual activity, etc.) or psychological violence (calling names, mocking, gossiping, someone shouted at them very strongly, threatened him, the child felt rejected by a loved him as a person) (yes/no)
4. Child has witnessed physical or mental violence against another person (yes/no)
5. Child has experienced the death of someone close to him (yes/no)
6. There were severe problems in the family (quarrels, conflicts, breakups, alcohol problem, other addictions, emotional problems of family members, etc.) (yes/no)
7. Child was separated from one of the parents for many days (yes/no)
8. Serious problems with grades at school (yes/no)
9. Another very stressful event (yes/no)

Evaluation of all the salivary biomarkers was performed according to subgroups SE1 and SE0.

### 2.5. Anthropometric Parameters

Body height, weight, waist, and hip circumferences were evaluated. Body height in a standing position was measured with the SECA 216 wall-mounted stadiometer with an accuracy of 0.1 cm. Body mass was recorded in lightweight clothing on a digital scale with an accuracy of 0.1 kg. Waist circumference was determined halfway between the lower edge of the rib arch and the upper iliac crest using metric tape [38]. Hip circumference was measured at a level parallel to the floor, at the largest circumference of the buttocks using metric tape [38]. All subjects were assessed for body mass index (BMI) according to the formula [39] BMI = weight (kg)/height^2^ (m^2^) with cut-offs adopted by the International Obesity Task Force (IOTF) to estimate normal or abnormal weight according to age.

### 2.6. Salivary Collection

Special attention was paid to standardize collection. All participants were referred to the medical school offices between 9 : 00 and 10 : 00 a.m. for examination and saliva sampling, performed by the same qualified dentist (E.P.).

Unstimulated whole saliva was collected according to a previously described methodology [40, 41]. Recommendations neither to eat for one hour before the examination nor to undergo any medical or oral hygiene procedures had been given prior to the visit. For saliva collection, children were asked to spit unstimulated saliva into a sterile container for 2 minutes, which was discarded, followed by another collection in a separate flask for 10 minutes. Children were asked to focus on spitting and limiting other activities while leaning forward in a seated position during the examination. Immediately after collection, the samples were centrifuged; the separated supernatant was first frozen at -20°C and then at -80°C until further processing.

### 2.7. Cortisol

The concentration of free cortisol in the saliva was quantitatively determined by ELISA using the ELISA Kit DES6611 for the in vitro diagnostic (IVD) (Demeditec Diagnostics GmbH, Kiel, Germany). The standard curve ranged from 0 to 30 ng/ml, the intra- and interassay variability coefficients were assessed to be below 6%, and the standard curve was statistically significant (*r*^2^ = 0.998, *p* < 0.001) [42, 43]. Cortisol levels are expressed in ng/mL.

### 2.8. Salivary Alpha-Amylase (sAA)

The sAA concentrations were quantitatively determined by ELISA using the ELISA Kit DEEQ6231 for the in vitro diagnostic (IVD) (Demeditec Diagnostics GmbH, Kiel, Germany). The standard curve ranged from 0 to 500 U/ml, the intra- and interassay variability coefficients were assessed to be below 5%, and the standard curve was statistically significant (*r*^2^ = 0.982, *p* < 0.001) [44, 45]. sAA levels are expressed in U/mL.

### 2.9. Salivary Secretory Immunoglobulin A (sIgA)

Salivary IgA concentrations were quantitatively determined by the ELISA method using the ELISA Kit DEXK276 for the in vitro diagnostic (IVD) (Demeditec Diagnostics GmbH, Kiel, Germany). The standard curve ranged from 0 to 400 *μ*g/ml, the intra- and interassay variability coefficients were assessed to be below 5%, and the standard curve was statistically significant (*r*^2^ = 0.995, *p* < 0.001) [2, 46]. Levels of sIgA are expressed in *μ*g/mL.

### 2.10. Opiorphin (OPI)

The quantification of OPI in saliva was performed using a commercial enzyme immunoassay (ELISA test for measuring human opiorphin cat. no. EH1927, FineTest, Wuhan, Hubei, China) according to the manufacturer's instructions. The measuring range of the kit was 0.156–10 ng/ml and sensitivity 0.094 ng/ml. The intraplatelet coefficient of variation was <8% with an interplatelet variation coefficient of <10%, and the standard curve was statistically significant (*r*^2^ = 0.985, *p* < 0.001). OPI levels are expressed in ng/mL.

All ELISA tests were performed according to the manufacturer's instructions, without any modification. All samples and standards were run in duplicates, and the mean value of the two assays was used for statistical evaluation. Optical density was read with a spectrophotometric plate reader (Asys UVM 340 Microplate Reader from Biochrom Ltd., Cambridge, UK) for a wavelength of 450 nm ± 10 nm. A four-parameter algorithm (4 parameters logistic) was used to assess concentration in the tested samples. All tests were performed by an investigator blinded to clinical data and the status of the samples (group affiliation).

Cortisol, sAA, and IgA were tested with validated *in vitro* diagnostic tests certified by CE IVD certificates. Measurements were selected only at the quantification level, and when two technical replicates were obtained, it was detected with an acceptable difference of <10%.

We used all the obtained nominal measurements for the calculations, treating them as individual natural variability, as we did not observe extreme deviations in results. We have not adopted any arbitrary cut-off thresholds to distinguish between the correct and the abnormal levels, mainly due to the lack of diagnostic standards for the examined children age group.

### 2.11. Statistical Analysis

Statistical analyses were conducted in Statistica v13.3 (StatSoft, Poland). Normality of distribution was assessed with the Shapiro-Wilk test, and equality of variances was checked using Levene's test. Due to the lack of normality for most of the variables, more restrictive nonparametric tests were used. Comparison of two unpaired groups was performed using the Mann–Whitney *U* test. Spearman's rank correlation was used to detect the relationship between variables. In order to rule out interfering variables that may be biasing the correlation results, we used covariance analysis (a combination of analysis of variance and regression (ANCOVA)) [47] to check the effect of the interaction of qualitative variables (age, gender, being overweight, stress life event-interfering variables) on the concentration level of the studied proteins: cortisol, sIgA, sAA, and OPI. A *p* value of <0.05 was considered statistically significant.

## 3. Results

### 3.1. Sample

**Figure 2:**
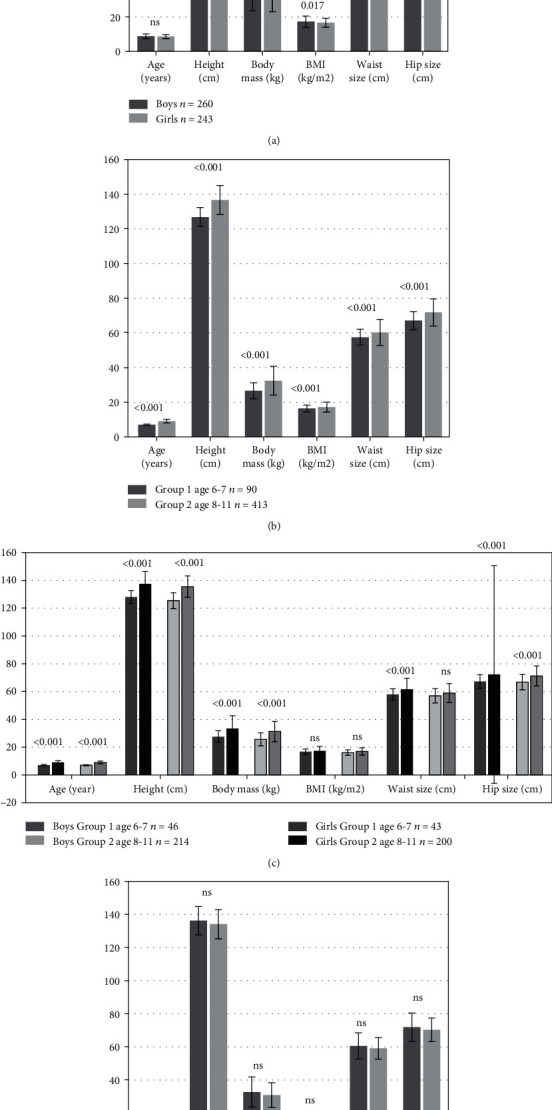
Anthropometric parameters according to (a) sex, (b) age group, (c) age group and sex, and (d) prior stress events (SE0 vs. SE1). One bar graph for each comparison (four graphs in total). Mean ± standard deviation and *p* value with statistical difference marked in values. *n*: number of examined children; ns: not significant value in statistical analysis; sIgA: salivary IgA; sAA: salivary alpha-amylase; OPI: opiorphin; SE1: group of children with stressful experience in the past; SE0: group of children without stressful experience in the past.

The final sample consisted of 503 children (Figure 1), 6-11 years old, (mean age 8.7 ± 1.3), including 260 boys (51.7%) and 243 girls (48.3%), with no statistical difference between mean age (8.8 ± 1.4) vs. (8.6 ± 1.2) (*p* > 0.05, Mann–Whitney *U* test), respectively. Not surprisingly, the anthropological measurements of body weight, height, BMI, and waist size were significantly different according to age and gender (*p* < 0.05, Welch test). Children from both groups were Caucasians of Polish origin attending a public school for primary education. There were no differences between confessed religions (mainly catholic), domestic-owned animals (mainly dogs), or living places. The MINI-KID questionnaire excluded children at risk of mental disturbances, and the TESI questionnaire included children who experienced stressful events. More than half reported a stressful experience in the past and feeling low mood during the past two weeks (group SE1 *n* = 411, 81.7%). The main characteristics of the sample are summarized in Figure 2.

### 3.2. Salivary Biomarkers

**Figure 3:**
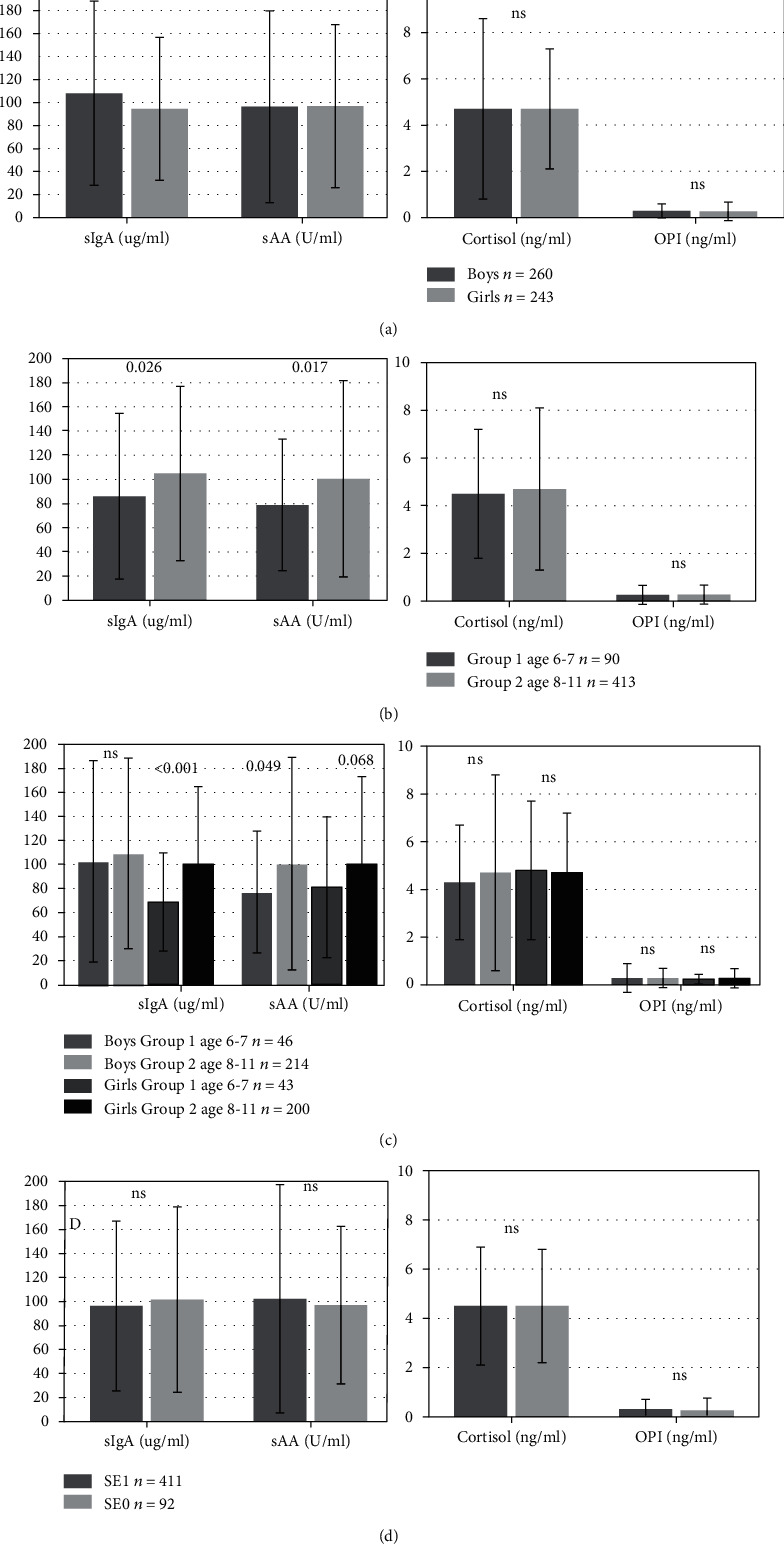
Salivary parameters according to (a) sex, (b) age group, (c) age group and sex, and (d) prior stress events (SE0 vs. SE1). One bar graph for each comparison (four graphs in total). Mean ± standard deviation and *p* value with statistical difference marked in values. *n*: number of examined children; ns: not significant value in statistical analysis; sIgA: salivary IgA; sAA: salivary alpha-amylase; OPI: opiorphin; SE1: group of children with stressful experience in the past; SE0: group of children without stressful experience in the past.

**Figure 4:**
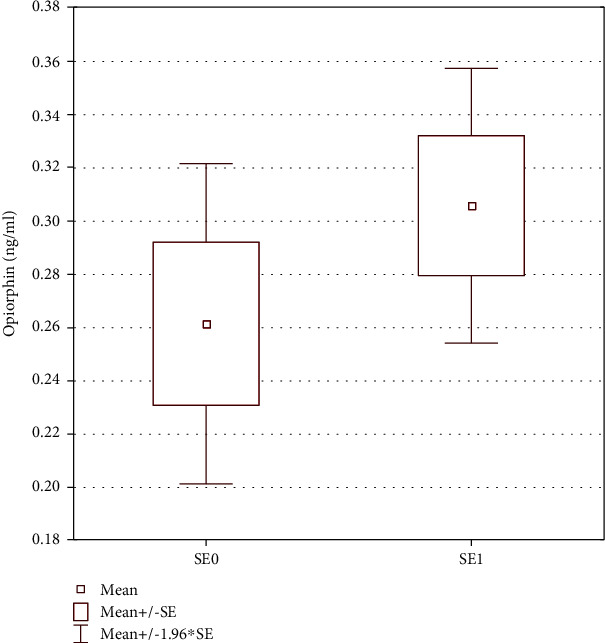
Comparison of salivary opiorphin (mean ± standard deviation). SE0: children free of stressful experiences in the past; SE1: children with stressful experiences in the past. Children from the SE1 group showed higher salivary concentration of this peptide. *p* value with statistical correlation, *p* < 0.05.

Salivary measurements are shown in Figure 3. In unstimulated saliva, sIgA showed the most significant difference among children according to sex and age (*p* < 0.038 and *p* < 0.026, respectively). A significant difference (*p* < 0.017) was observed for sAA, found in higher concentration in group 2 aged 8-11 (*p* < 0.017), especially for boys (*p* < 0.049). No differences in the levels of cortisol and OPI were detected depending on sex (*p* = 0.901 and *p* = 0.721, respectively) and age (*p* = 0.644 and *p* = 0.784, respectively) or any anthropometric parameter. However, OPI was detected in statistically significant higher concentration in children having experienced distressing events (group SE1, *p* < 0.031) (see Figure 4). The rest of salivary biomarkers sIgA, sAA, or cortisol did not change (*p* = 0.705, *p* = 0.711, and *p* = 0.491, respectively) for this condition.

### 3.3. Correlations

Correlations are shown in Table 2. Spearman analysis revealed the correlation between sIgA and all measured anthropometric parameters, such as age, height, body mass, BMI, waist, and hip circumferences (*p* < 0.05) for all children (Table 2). A similar correlation between sAA and selective measured anthropometric parameters, such as age, height, and hip size in the total group and body mass with waist size in girls aged 6-11 (*p* < 0.05), was evidenced. OPI showed a significant correlation between age and height in group 1, together with sAA in the whole group of boys (*p* < 0.05). OPI was the sole marker positively correlated and sAA negatively correlated with children declaring prior distressing experiences and low mood (*p* < 0.05) (Figure 4). There was no significant correlation neither for cortisol according to age and sex differences nor for the other salivary biomarkers (*p* > 0.05) (Table 2).

### 3.4. Covariance Analysis

Four covariance models (ANCOVA) (shown in Table 3) were built ((1) sIgA, (2) sAA, (3) cortisol, and (4) opiorphin) for testing the effect of interaction between the following clinical factors: (1) age divided into younger and older children, (2) sex divided into boys and girls, (3) BMI divided into normal and abnormal indexes with cut-offs adopted by the International Obesity Task Force (IOTF), and (4) stressful experiences SE1 (yes) or SE0 (no). Statistical analysis showed that sAA and OPI were not dependent on variables such as age, gender, body weight (normal/obesity), and past stressful events, but sIgA was dependent on the age of children (*p* < 0.001) as cortisol on body mass index (*p* < 0.046).

## 4. Discussion

The main results of this study are significant differences in sIgA and sAA among children depending on age and gender. Correlations between salivary immune biomarkers sIgA, sAA, age, sex, and parameters of developmental status were evidenced. Opiorphin was detected in statistically higher concentrations in children reporting previous experiences of stressful events.

In the present study, concentrations of sIgA and sAA were correlated to age and anthropometric parameters. The concentration of sAA showed significant developmental differences in particular for boys, especially for those of the older age group. Other studies focusing on human development have shown that salivary components and salivary gland function may alter with age [48]. The secretion of sAA changes with age, from low levels in neonates to levels similar to those of adults during adolescence; later, sAA remains stable throughout adulthood, even in old age. The second aspect of the present study concerning how mental stress may influence the secretion of sAA is also interesting. This salivary enzyme is mainly produced by the parotid glands but reflects the activation of the autonomic nervous system. Both physiological and psychological factors stimulate *α*-amylase release during stress [49, 50], and different modes of stimulation (taste stimulation for parotid gland activity, psychological stress, and physical effort) result in different patterns of sAA release [51–53].

In our study, the increase in sAA levels with age and physical developmental changes suggests a possible application of saliva biochemical analyses to monitor young children's health. Saliva has been increasingly suggested as a good, easily obtainable sample to assess the health status. In this study, the inverse correlation between mental stress among children in group SE1 and sAA was relatively weak. The covariance models (ANCOVA) testing the effects of interaction between clinical factors and salivary parameters did not indicate sAA as a significant one.

Similarly to sAA, sIgA levels in the present study were different depending on age. This finding is consistent with previous findings reported by other researchers among children and adult subjects [54–58]. However, none of these studies reported significant differences between adult men and women or boys and girls. Male subjects had higher sIgA levels than females. The reason for this sex difference may be found in salivary secretion level usually higher in males than females [59, 60]. Hormones might also play a role, although children under 11 years are usually not under hormonal influence, at least sex steroid impregnation. The present study included only healthy children in prehormonal development, confirming the age-sex-related differences in IgA secretion. An interesting observation is that maximum secretion may be reached in elderly age, e.g., >80 years old [54, 56], suggesting that sIgA is not an aging-related event but rather a result of disease, including exposure to oral pathogens and subsequent pharmacotherapies [61, 62]. Indeed, the correlation evidenced here between sIgA and cortisol only among girls supports previous research highlighting the relationship between sex, HPA axis, and immunocompetency [27, 63]. Female patients might be more prone to the effects of stress in ages under 11 years old. Studies focusing on adult women, indicating that they are more sensitive to stress events and display higher anxiety levels when visiting the medical/dental facilities than men, may support this hypothesis [64–66]. The increase in sIgA among girls and correlation with age confirmed in the covariance model advocates that prophylactic mental health programs early in childhood might be directed towards girls because of their higher reactivity to mental stress in adult age.

OPI was the only salivary biomarker elevated in children who self-reported previous exposure to stress. Statistical analysis (ANCOVA test) showed that OPI levels were not dependent on age, sex, and body weight (normal/obesity), thus reinforcing the statistical link between OPI levels and past stressful events. Although declarative in nature, to our knowledge, no study to date has compared opiorphin to healthy children with and without self-declared past distressing events. OPI has been described as a potent endogenous antinociceptive [67] and stress-related peptide [28, 30, 67] in adults. Similarly, in female adolescents affected by eating disorders, salivary changes in OPI and sIgA were observed, suggesting a possible use of OPI as a neurobiological salivary biomarker of malnutrition [27]. The present results suggest that prepubertal children (<12 y.o.) might also have a measurable response to stress through OPI.

Therefore, further research specifically exploring the role of stress needs to be continued among such young individuals.

No evidence of specific reactive secretion differences was found between children for cortisol levels. Moreover, cortisol levels were unchanged among those who declared previous stressful events, suggesting a well-functioning HPA system. Cortisol levels, measured at the same time point as sIgA and sAA, did not show sex-related differences. However, the covariance models (ANCOVA) showed a dependence between BMI and salivary cortisol. This interrelation may suggest a dependent release from separate systems and individual diurnal settings [68, 69]. In the case of free cortisol, the daily fluctuation has to be taken into account, since in healthy adults, its concentration increases from 50% to 160% in the first 30 minutes after waking up [70]. In this study, no attempt to control the time that had elapsed after awakening in each individual was taken. We aimed at measuring differences in children subjected to a consistent routine in similar school conditions. In this study, the highest value for cortisol was 54 ng/ml, equal to 5.4 *μ*g/dl and 148.98 nmol/l, which is within normal range [71].

### 4.1. Limitations of the Study

In the present study, much attention was paid to collect a well-controlled cohort matched according to age and sex, with similar demographic features, representative of the general population. The group aged 8-11 was the largest, while the group aged 6-7 was lower in all chosen schools. A possible explanation for such unequal distribution is the overlap between school education and kindergarten for the youngest children. The study excluded children attending kindergartens and focused on those attending schools. Schools share a similar education system regarding rhythms, activities, and programs, which is not the case for kindergartens.

We also used a standardized instrument for evaluating the psychological stress among such young children with professional analysis performed by the child and adolescent specialists. However, the exposure to stress was only declarative, as evaluated by the TESI questionnaire. Neither further investigations of the nature of the stress nor assessment of the severity of stress was undertaken. We may also assume that the level of perceived stress for the same stressor may differ from person to person [72, 73]. Further studies designed for this purpose could give insights into the variations of assessed biomarkers with type and perceived level of stress.

Another possible bias of the study was the acute psychological stress that may occur during physical measurement and salivary sampling in the school area, despite all precautions taken. We also limited our explorations to a quartet of salivary biomarkers when a larger set of biomarkers would have provided additional valuable information, especially inflammatory biomarkers as well as oral health indicators.

## 5. Conclusions

Results of the present study among healthy children indicate that sIgA and sAA can be used as possible sex- and age-related biomarkers in children 6-12 y.o., which is not the case for free cortisol and OPI. The importance of sIgA and sAA findings may support monitoring the age-sex differences, especially in the prehormonal period of life.

However, OPI levels reflected previous exposure to stress, suggesting its evaluation of stress-related changes in prepubertal children. Interactions between BMI and salivary cortisol release suggest the dependence of such secretion with abnormal body weight in obese children.

## Figures and Tables

**Table 1:** Inclusion and exclusion criteria for both groups.

Criteria for inclusion into the study group 1	Criteria for inclusion into the study group 2	Criteria for exclusion from study groups
Children of both girls and boys aged 6–7	Children of both girls and boys aged 8–11	Children of age <6 and >12 years both girls and boys
Lack of mental disorders—assessment with the use of MINI-KID questionnaire [36]	Lack of mental disorders—assessment with the use of MINI-KID questionnaire [36]	Children with mental disorders—assessment with the use of MINI-KID questionnaire [36] (e.g., coexisting schizophrenia, bipolar affective disorder, and serious somatic disorders)
Parent or legal guardian approval	Parent or legal guardian approval	Lack of acceptance from parents or legal guardian
Children without hereditary mental disorders (first-degree relatives)	Children without hereditary mental disorders (first-degree relatives)	Children with disorders of the central nervous system (e.g., epilepsy, serious injuries, and CNS infections)
Healthy intellectual and physical ability to collaborate during the study	Intellectual and physical ability to collaborate during the study	Chronic pharmacotherapy, hormonotherapy, dietary supplements
Good oral health	Good oral health	Children attending urgent or nonroutine dental treatment prone to alter salivary flow and composition orthodontic treatment

MINI-KID: Mini International Neuropsychiatric Interview for Kids questionnaire; CNS: central nervous system.

**Table 2:** Spearman's rank order correlations between total salivary parameters, divided by groups (young children group 1 and older children group 2), boys and girls.

Variables	Spearman's rank order correlations				
	Total (age 6-11) (*n* = 503)	Group 1 (age 6-7) (*n* = 90)	Group 2 (age 8-11) (*n* = 413)	Boys (age 6-11) (*n* = 260)	Girls (age 6-11) (*n* = 243)
	*r*_*s*_	*r*_*s*_	*r*_*s*_	*r*_*s*_	*r*_*s*_
sIgA vs. age	0.19^∗^	-0.21	0.17^∗^	0.16^∗^	0.22^∗^
sIgA vs. height	0.24^∗^	0.03	0.24^∗^	0.22^∗^	0.25^∗^
sIgA vs. body mass	0.22^∗^	-0.05	0.24^∗^	0.21^∗^	0.22^∗^
sIgA vs. BMI	0.12^∗^	-0.06	0.15^∗^	0.11	0.13^∗^
sIgA vs. waist size	0.15^∗^	-0.08	0.17^∗^	0.14^∗^	0.15^∗^
sIgA vs. hip size	0.21^∗^	0.02	0.21^∗^	0.19^∗^	0.22^∗^
sIgA vs. cortisol					0.14^∗^
sAA vs. age	0.11^∗^	0.12	0.03	0.10	0.12
sAA vs. height	0.08^∗^	-0.09	0.05	0.05	0.12
sAA vs. body mass	0.10	-0.03	0.07	0.07	0.13^∗^
sAA vs. BMI	0.09	-0.01	0.09	0.06	0.12
sAA vs. waist size	0.08	-0.03	0.08	0.04	0.13^∗^
sAA vs. hip size	0.10^∗^	0.04	0.08	0.08	0.12
sAA vs. SE1	-0.10^∗^				
Cortisol vs. age	0.001	0.03	-0.05	0.00	0.01
Cortisol vs. height	-0.02	-0.03	-0.03	-0.02	-0.02
Cortisol vs. body mass	-0.01	0.01	-0.02	-0.02	0.00
Cortisol vs. BMI	-0.02	0.02	-0.03	-0.03	0.00
Cortisol vs. waist size	-0.01	-0.01	0.00	0.00	-0.01
Cortisol vs. hip size	-0.02	0.001	-0.03	-0.07	0.02
OPI vs. age	0.04	0.32^∗^	0.04	0.02	0.06
OPI vs. height	0.10^∗^	0.27^∗^	0.10	0.08	0.11
OPI vs. body mass	0.09	0.04	0.09	0.06	0.12
OPI vs. BMI	0.05	-0.11	0.07	0.00	0.10
OPI vs. waist size	0.10^∗^	0.11	0.09	0.08	0.12
OPI vs. hip size	0.05	-0.01	0.06	0.02	0.08
OPI vs. sAA				0.15^∗^	
OPI vs. SE1	0.11^∗^				

sIgA: salivary IgA; sAA: salivary alpha-amylase; OPI: opiorphin; SE1: children with distressing events in the past; BMI: body mass index; vs.: versus; *n*: number of patients; ns: statistically nonsignificant. ^∗^*p* value with statistical correlation, *p* < 0.05.

**Table 3:** The covariance models (ANCOVA) testing effect of interaction between clinical factors and salivary parameters.

Variable	Model 1 (sIgA)		Model 2 (sAA)		Model 3 (cortisol)		Model 4 (OPI)	
	*F*	*p* value	*F*	*p* value	*F*	*p* value	*F*	*p* value
(1) Age: group 1 (age 6-7) and group 2 (age 8-11)	10.964	**0.001**	2.057	0.152	0.032	0.858	0.170	0.681
(2) Sex: boys and girls	1.770	0.184	0.218	0.641	0.430	0.512	0.337	0.562
(3) BMI: normal and abnormal	1.929	0.166	2.468	0.117	3.996	**0.046**	0.063	0.802
(4) Stressful experiences: SE1(yes) and SE0 (no)	2.246	0.135	0.856	0.356	0.660	0.417	0.004	0.952
1∗2	1.735	0.188	0.579	0.447	0.597	0.440	0.234	0.629
1∗3	1.191	0.276	0.413	0.521	0.046	0.831	0.232	0.631
2∗3	0.007	0.935	2.090	0.149	0.195	0.659	0.035	0.851
1∗4	0.001	0.977	0.000	1.000	0.943	0.332	3.122	0.078
2∗4	0.519	0.472	0.109	0.742	0.507	0.477	0.063	0.803
3∗4	0.837	0.361	1.699	0.193	0.478	0.490	0.924	0.337
1∗2∗3	0.723	0.396	3.679	0.056	1.252	0.264	0.020	0.889
1∗2∗4	2.958	0.086	0.861	0.354	0.169	0.681	0.187	0.666
1∗3∗4	0.428	0.513	0.131	0.718	1.246	0.265	0.055	0.814
2∗3∗4	1.196	0.275	0.000	0.987	2.501	0.115	0.789	0.375
1∗2∗3∗4	2.020	0.156	0.714	0.399	3.208	0.074	0.646	0.422

sIgA: salivary IgA; sAA: salivary alpha-amylase; OPI: opiorphin; SE1: children with distressing events in the past; SE0: children without distressing events in the past; BMI: body mass index. Significant *p* value effect was marked (*p* < 0.05); body mass index (BMI) cut-offs were adopted by the International Obesity Task Force (IOTF).

## Data Availability

The datasets generated for this study are available on request to the corresponding author.

## Supplementary Materials

Supplementary 1 Supplementary Materials Tables S1, S2, and S3. Table S1: descriptive statistics of anthropometric and salivary parameters for the whole group according to female and male sex distribution. Mean ± standard deviation, median (Min–Max), and ^∗^*p* value with statistical difference. *n*: number of examined children; ns: not significant value in statistical analysis; vs.: versus; sIgA: salivary IgA; sAA: salivary alpha-amylase; OPI: opiorphin. Table S2: descriptive statistics of anthropometric and salivary parameters for the whole group in age groups (group 1 children between 6 and 7 years old and group 2 children between 8 and 11 years old). Mean ± standard deviation, median (Min–Max), and ^∗^*p* value with statistical difference. *n*: number of examined children; ns: not significant value in statistical analysis; vs.: versus; sIgA: salivary IgA; sAA: salivary alpha-amylase; OPI: opiorphin. Table S3: descriptive statistics of anthropometric and salivary parameters for the whole group in female and male subgroups (group 1 only girls or boys aged between 6 and 7 years old and group 2 only girls or boys aged between 8 and 11 years old). Mean ± standard deviation, median (Min–Max), and ^∗^*p* value with statistical difference. *n*: number of examined children; ns: not significant value in statistical analysis; vs.: versus; sIgA: salivary IgA; sAA: salivary alpha-amylase; OPI: opiorphin.

Supplementary 2 Supplementary Material: Traumatic Events Screening Inventory-Parent Report Revised (Polish language version).
