# Supplementary material for: Salivary Biomarkers (Opiorphin, Cortisol, Amylase, and IgA) Related to Age, Sex, and Stress Perception in a Prospective Cohort of Healthy Schoolchildren
Source: Mediators Inflamm. 2021 Nov 12;2021:3639441. doi: 10.1155/2021/3639441 (PMC8801773; doi:10.1155/2021/3639441)
Supplement: Supplementary 1 — Supplementary Materials Tables S1, S2, and S3. Table S1: descriptive statistics of anthropometric and salivary parameters for the whole group according to female and male sex distribution. Mean ± standard deviation, median (Min–Max), and ∗p value with statistical difference. n: number of examined children; ns: not significant value in statistical analysis; vs.: versus; sIgA: salivary IgA; sAA: salivary alpha-amylase; OPI: opiorphin. Table S2: descriptive statistics of anthropometric and salivary parameters for the whole group in age groups (group 1 children between 6 and 7 years old and group 2 children between 8 and 11 years old). Mean ± standard deviation, median (Min–Max), and ∗p value with statistical difference. n: number of examined children; ns: not significant value in statistical analysis; vs.: versus; sIgA: salivary IgA; sAA: salivary alpha-amylase; OPI: opiorphin. Table S3: descriptive statistics of anthropometric and salivary parameters for the whole group in female and male subgroups (group 1 only girls or boys aged between 6 and 7 years old and group 2 only girls or boys aged between 8 and 11 years old). Mean ± standard deviation, median (Min–Max), and ∗p value with statistical difference. n: number of examined children; ns: not significant value in statistical analysis; vs.: versus; sIgA: salivary IgA; sAA: salivary alpha-amylase; OPI: opiorphin. [file 3639441.f1.docx]

Table S1: Descriptive statistics of anthropometric and salivary parameters for the whole group according to female and male sex distribution.

| Group  Parameter | Boys  n=260 | Girls  n=243 | Total group  n=503 | p-value  boys vs. girls |
| --- | --- | --- | --- | --- |
| Age [years] | 8.8±1.4  **8.7** (6-11.11) | 8.6±1.2  **8.6** (6-11.6) | 8.7±1.3  **8.6** (6-11.11) | ns |
| Height [cm] | 135.8±9.1  **135** (115-166) | 133.8±8.3  **133.6** (115-159) | 134.8±8.8  **134** (115-166) | 0.008* |
| Body mass [kg] | 32.4±8.8  **31** (18.9-88.6) | 30.3±7.2  **28.8** (18.9-59.6) | 31.4±8.1  **29.6** (18.9-88.6) | 0.003* |
| BMI [kg/m^2^] | 17.3±3.3  **16.5** (12.6-34.1) | 16.7±2.6  **16** (12.8-26) | 17±2.8  **16.3** (12.6-34.1) | 0.017* |
| Waist size [cm] | 61±7.5  **59** (38-95) | 58.5±6.6  **57** (46-81) | 59.8±7.2  **58** (38-95) | <0.001* |
| Hip size [cm] | 71.4±8  **70** (50-114) | 70.5±7.2  **70** (56-97) | 71±7.6  **70** (50-114) | ns |
| sIgA  [ug/ml] | 108.1±80.1  **81** (1.1-450) | 94.6±62.2  **80.1** (16-380) | 101.6±72.3  **80.1** (1.1-450) | 0.038* |
| sAA  [U/ml] | 96.4±83.4  **81.2** (3-959) | 96.9±70.8  79.2 (1.3-598) | 96.6±71.8  **81.8** (1.3-959) | ns |
| Cortisol [ng/ml] | 4.7±3.9  **3.8** (1.1-54) | 4.7±2.6  **3.9** (0.2-17) | 4.7±3.3  **3.6** (0.2-54) | ns |
| OPI [ng/ml] | 0.29±0.3  **0.16** (0.02-4.2) | 0.27±0.4  0.15 (0.004-2.9) | 0.28±0.4  **0.15** (0-4.2) | ns |

* Mean±Standard Deviation, **Median** (Min-Max), ** p-value with statistical difference

n – number of examined children, ns – not significant value in statistical analysis, vs. – versus, sIgA-salivary IgA, sAA-salivary alpha-amylase, OPI-opiorphin

Table S2: Descriptive statistics of anthropometric and salivary parameters for the whole group in age groups (group 1 children between 6-7 years old, group 2 children between 8-11 years old).

| **Group**  **Parameter** | Group 1  age 6-7  n=90 | Group 2  age 8-11  n=413 | p-value  group 1 vs.  group 2 | Children with stressful experiences in the past  SE1  n=411 | Children free of stressful experiences in the past  SE0  n=92 | p-value  group SE1 vs.  SE0 |
| --- | --- | --- | --- | --- | --- | --- |
| Age [years] | 7±0.4  **7.1** (6-7.5) | 9.1±1.1  **8.9** (8-11.9) | <0.001* | 9.0±1.4  **8.9** (6.4-11.9) | 8.7±1.2  **8.5** (6.1-11.9) | ns |
| Height [cm] | 126.8±5.5  **126** (115-139) | 136.6±8.4  **135** (117-166) | <0.001* | 136.2±8.6  **134** (120-161) | 134.1±8.8  **134** (116-166) | ns |
| Body mass [kg] | 26.6±4.6  **26** (19-39) | 32.4±8.3  **30.5** (19-89) | <0.001* | 32.7±9.1  **34.4** (19-89) | 30.8±7.5  **30** (19-62) | ns |
| BMI [kg/m^2^] | 16.4±2  **16** (13-22) | 17.2±2.9  **16.4** (13-34.1) | 0.020* | 17.4±3.3  **17** (13-34.1) | 16.9±2.5  **16.3** (13.3-31.4) | ns |
| Waist size [cm] | 57.5±4.6  **55** (49-72) | 60.3±7.5  **59** (38-95) | <0.001* | 60.6±7.9  **58** (49-95) | 59.1±6.5  **58** (46-92) | ns |
| Hip size [cm] | 67.1±5.2  **67** (57-82) | 71.8±7.8  **70** (50-114) | <0.001* | 71.9±8.6  **70** (53-114) | 70.3±7.1  **70** (56-100) | ns |
| sIgA  [ug/ml] | 86±68.6  **63.5** (16.2-450.3) | 104.9±72.1  **84.8** (1.1-420) | 0.026* | 96.2±70.8  **75** (15.3-361) | 101.6±77.3  **78** (1.1-450.1) | ns |
| sAA  [U/ml] | 78.9±54.4  **69** (3.1-321.1) | 100.5±81.2  **89.2** (1.3-959.3) | 0.017* | 102.2±95.1  **79.3** (3-959.3) | 96.9±65.6  **90** (3.1-591) | ns |
| Cortisol [ng/ml] | 4.5±2.7  **3.7** (1.4-16) | 4.7±3.4  **3.9** (0.2 54) | ns | 4.5±2.4  **3.8** (1-17) | 4.5±2.3  **3.9** (1.3-16.1) | ns |
| OPI [ng/ml] | 0.27±0.4  **0.2** (0-3.2) | 0.28±0.4  **0.1** (0-4.2) | ns | 0.31±0.4  **0.2** (0.009-2) | 0.26±0.5  **0.1** (0.004-3.2) | 0.031* |

* Mean±Standard Deviation, **Median** (Min-Max), *p-value with statistical difference

n – number of examined children, ns – not significant value in statistical analysis, vs. – versus, sIgA-salivary IgA, sAA-salivary alpha-amylase, OPI-opiorphin

Table S3: Descriptive statistics of anthropometric and salivary parameters for the whole group in female and male subgroups (group 1 only girls or boys aged between 6-7 years old, group 2 only girls or boys aged between 8-11 years old).

| Group  Parameter | Boys  Group 1  age 6-7  n=46 | Boys  Group 2  age 8-11  n=214 | p-value  group 1 vs.  group 2  (only boys) | Girls  Group 1  age 6-7  n=43 | Girls  Group 2  age 8-11  n=200 | p-value  group 1 vs.  group 2  (only girls) |
| --- | --- | --- | --- | --- | --- | --- |
| Age [years] | 7.0±0.5  **7** (6-7.5) | 9.1±1.2  **8.9** (8-11.9) | <0.001* | 7±0.4  **7** (6-7.5) | 9±1  9 (8-11.7) | <0.001* |
| Height [cm] | 128.0±4.7  **127** (115-139) | 137.5±8.9  **136** (120-166) | <0.001* | 125.5±5.7  **125** (115-138) | 135.5±7.7  **135** (117-159) | <0.001* |
| Body mass [kg] | 27.5±4.3  **26** (20-39) | 33.5±9.1  **32** (19-89) | <0.001* | 25.6±4.7  **25** (19-38.4) | 31.3±7.3  **30** (19.6-59.6) | <0.001* |
| BMI [kg/m^2^] | 16.7±2  **16** (13-21) | 17.4±3.1  **17** (13-34) | ns | 16.1±2  **16** (13-20) | 16.9±2.7  **16.2** (12.8-26) | ns |
| Waist size [cm] | 57.9±4.1  **57** (52-72) | 61.7±7.9  **60** (38-75) | <0.001* | 57±5.2  **56** (49-69) | 58.9±6.8  **57** (46-81) | ns |
| Hip size [cm] | 67.3±4.9  **68** (57-82) | 72.3±78.3  **71** (50-114) | <0.001* | 66.9±5.6  **66** (58-79) | 71.3±7.2  **70** (56-97) | <0.001* |
| sIgA  [ug/ml] | 102.5±84  **71** (16-450) | 109.2±79.4  **86** (1.1-421) | ns | 68.7±41  **58** (16.2-200) | 100.3±64.5  **84.4** (16-380.3) | <0.001* |
| sAA  [U/ml] | 77.0±50.8  **65** (3-247) | 100.7±88.5  **90** (4-959) | 0.049* | 81±58.6  **72** (5.1-321) | 100.4±72.9  **85** (1.3-590.8) | 0.068 |
| Cortisol [ng/ml] | 4.3±2.4  **3.8** (1.4-16) | 4.7±4.1  **4** (1-54) | ns | 4.8±2.9  **4** (1.7-13.5) | 4.7±2.5  **3.9** (0.2-17) | ns |
| OPI [ng/ml] | 0.29±0.6  **0.2** (0.04-3.2) | 0.29±0.4  **0.2** (0.004-4.18) | ns | 0.24±0.2  **0.2** (0-0.7) | 0.28±0.4  **0.1** (0-2.9) | ns |

Mean±Standard Deviation, **Median** (Min-Max), *p-value with statistical difference

n – number of examined children, ns – not significant value in statistical analysis, vs. – versus, sIgA-salivary IgA, sAA-salivary alpha-amylase, OPI-opiorphin
