# Supplementary material for: Salivary Biomarkers (Opiorphin, Cortisol, Amylase, and IgA) Related to Age, Sex, and Stress Perception in a Prospective Cohort of Healthy Schoolchildren
Source: Mediators Inflamm. 2021 Nov 12;2021:3639441. doi: 10.1155/2021/3639441 (PMC8801773; doi:10.1155/2021/3639441)
Supplement: Supplementary 2 — Supplementary Material: Traumatic Events Screening Inventory-Parent Report Revised (Polish language version). [file 3639441.f2.docx]

***TRAUMATIC EVENTS SCREENING INVENTORY-PARENT REPORT REVISED (TESI-PRR)***

**OFFICE ONLY** ID:_________ Respondent: __________ Times @ Clinic_______ Date:______________

Assessor:_________________________ Vscale___________ VP1: ❑ Y ❑ N VP2: ❑ Y ❑ N VP3: ❑ Y ❑ N

**TRAUMATIC EVENTS SCREENING INVENTORY- PARENT REPORT REVISED**

Dzieci mogą doświadczać stresujących wydarzeń, które mogą wpływać na ich zdrowie i samopoczucie. Wskaż proszę, *czy* Twoje dziecko doświadczyło któregokolwiek z tych potencjalnie stresujących wydarzeń, odpowiadając na pytania w szarych polach. Jeśli odpowiedź brzmi TAK, odpowiedz proszę na kolejne pytania. Jeśli odpowiedź brzmi NIE, przejdź do kolejnego, zaznaczonego na szaro pytania.

Jeśli masz jakiekolwiek pytanie lub uwagę dotyczącą któregokolwiek z pytań, z przyjemnością z Tobą o nim porozmawiamy.

| PRZYKŁAD (*instrukcje pisane są kursywą*) | ❑ Tak  ❑ Nie  ❑ Nie wiem |
| --- | --- |
| 1. **Czy Twoje dziecko było kiedykolwiek u lekarza?** (*Zaznacz odpowiedź w następnej kolumnie. Jeśli odpowiedziałeś TAK, odpowiedz na pytania poniżej*) |  |
| **Jeśli TAK – W jakim wieku było dziecko?**  **Pierwszy raz ______ Ostatni raz ______ Najbardziej stresujący ______**  *Wiek Twojego dziecka podczas ostatniej wizyty*  *Wiek Twojego dziecka, kiedy po raz pierwszy było u doktora (nawet, jeśli tego nie pamięta)*  *Wiek Twojego dziecka podczas najbardziej dla niego stresującej wizyty (według Twojej opinii)*  **Czy Twoje dziecko było pod silnym wpływem co najmniej jednego z tych zdarzeń? ❑ Tak ❑ Nie ❑ Nie wiem**  (*Poprzez określenie “pod silnym wpływem” mamy na myśl: czy Twoje dziecko wydawało się: a) bardzo przerażone; b) zdezorientowane lub bezradne; c) bardzo zszokowane lub wstrząśnięte; d) miało problem z powrotem do normalnego dla niego zachowania lub samopoczucia, kiedy było już po wszystkim, LUB e) zachowywało się wyraźnie inaczej kiedy było po wszystkim.)* |  |
|  |  |
| 1.1 Czy Twoje dziecko kiedykolwiek **brało udział** w poważnym wypadku, w którym ktoś mógł zostać (lub był) ranny lub mógł zginąć (zginął) (np. poważny wypadek samochodowy lub rowerowy, upadek, pożar; zdarzenie, w którym ktoś został poparzony, był bliski utonięcia lub utonął lub odniósł poważny uraz podczas uprawiania sportu) | ❑ Tak  ❑ Nie  ❑ Nie wiem |
| Jeśli TAK – Określ rodzaj wypadku(ów): _________________________________________________________________________________________________  Relacja ofiary z Twoim dzieckiem:__________________________________ Czy ktoś zginął? ❑ Tak ❑ Nie ❑ Nie wiem  Ile lat miało Twoje dziecko? Po raz pierwszy ________ Ostatni raz ________ Najbardziej stresujący ________  Czy Twoje dziecko było pod silnym wpływem co najmniej jednego z tych zdarzeń? ❑ Tak ❑ Nie ❑ Nie wiem |  |
| 1.2 Czy Twoje dziecko kiedykolwiek **widziało** poważny wypadek, w którym ktoś mógł zostać (lub był) ranny lub mógł zginąć (zginął) (np. poważny wypadek samochodowy lub rowerowy, upadek, pożar; zdarzenie, w którym ktoś został poparzony, był bliski utonięcia lub utonął lub odniósł poważny uraz podczas uprawiania sportu) | ❑ Tak  ❑ Nie  ❑ Nie wiem |
| Jeśli TAK – Określ rodzaj wypadku(ów): _________________________________________________________________________________________________  Relacja ofiary z Twoim dzieckiem:__________________________________ Czy ktoś zginął? ❑ Tak ❑ Nie ❑ Nie wiem  Ile lat miało Twoje dziecko? Po raz pierwszy ________ Ostatni raz ________ Najbardziej stresujący ________  Czy Twoje dziecko było pod silnym wpływem co najmniej jednego z tych zdarzeń? ❑ Tak ❑ Nie ❑ Nie wiem |  |

| 1.3 Czy Twoje dziecko przeżyło katastrofę naturalną, w której ktoś mógł zostać (lub został) ranny lub mógł zginąć (zginął) lub w której Twoja rodzina lub członkowie Twojej społeczności zaginęli lub musieli na zawsze opuścić swój dom (np. trąba powietrzna, huragan, pożar, trzęsienie ziemi)? | ❑ Tak  ❑ Nie  ❑ Nie wiem |
| --- | --- |
| Jeśli TAK – Określ rodzaj katastrofy: ________________________________________________ Czy ktoś zginął? ❑ Tak ❑ Nie ❑ Nie wiem  Ile lat miało Twoje dziecko? Po raz pierwszy ________ Ostatni raz ________ Najbardziej stresujący ________  Czy Twoje dziecko było pod silnym wpływem co najmniej jednego z tych zdarzeń? ❑ Tak ❑ Nie ❑ Nie wiem |  |
| 1.4 Czy Twoje dziecko kiedykolwiek doświadczyło sytuacji, w której ktoś mu bliski był poważnie chory lub został poważnie ranny? | ❑ Tak  ❑ Nie  ❑ Nie wiem |
| Jeśli TAK – Jaka była relacja tej osoby z Twoim dzieckiem?__________________________________  Ile lat miało Twoje dziecko? Po raz pierwszy ________ Ostatni raz ________ Najbardziej stresujący ________  Czy Twoje dziecko było pod silnym wpływem co najmniej jednego z tych zdarzeń? ❑ Tak ❑ Nie ❑ Nie wiem |  |
| 1.5 Czy Twoje dziecko kiedykolwiek doświadczyło śmierci kogoś mu bliskiego? | ❑ Tak  ❑ Nie  ❑ Nie wiem |
| Jeśli TAK – Jaka była relacja tej osoby z Twoim dzieckiem?__________________________________  Ile lat miało Twoje dziecko? Po raz pierwszy ________ Ostatni raz ________ Najbardziej stresujący ________  Czym była spowodowana śmierć? *(zaznacz wszystkie właściwe):* ❑ z przyczyn naturalnych ❑ choroba ❑ wypadek ❑ przemoc ❑ nieznana  Czy Twoje dziecko było pod silnym wpływem co najmniej jednego z tych zdarzeń? ❑ Tak ❑ Nie ❑ Nie wiem |  |
| 1.6 Czy Twoje dziecko przeszło kiedykolwiek poważne operacje lub cierpiało na chorobę zagrażającą życiu? Albo zajmował się nim ratownik medyczny na pogotowiu, lub spędziło noc w szpitalu ze względu na przebytą operację? | ❑ Tak  ❑ Nie  ❑ Nie wiem |
| Jeśli TAK – opisz _______________________________________________________________________________________________________________________  Ile lat miało Twoje dziecko? Po raz pierwszy ________ Ostatni raz ________ Najbardziej stresujący ________  Czy Twoje dziecko było pod silnym wpływem co najmniej jednego z tych zdarzeń? ❑ Tak ❑ Nie ❑ Nie wiem |  |
| 1.7 Czy Twoje dziecko było kiedykolwiek rozdzielone z Tobą lub inną osobą, która zapewniała dziecku miłość lub bezpieczeństwo, na więcej niż kilka dni LUB w bardzo stresujących okolicznościach? Na przykład przez opiekę zastępczą, ze względu na imigrację, wojnę, poważną chorobę lub pobyt w szpitalu. | ❑ Tak  ❑ Nie  ❑ Nie wiem |
| Jeśli TAK – Z kim dziecko zostało rozdzielone?________________________________________________________________________________________________  Ile lat miało Twoje dziecko? Po raz pierwszy ________ Ostatni raz ________ Najbardziej stresujący ________  Czy Twoje dziecko było pod silnym wpływem co najmniej jednego z tych zdarzeń? ❑ Tak ❑ Nie ❑ Nie wiem |  |
| 1.8 Czy kiedykolwiek ktoś bliski Twojemu dziecku próbował popełnić samobójstwo lub samookaleczał się? | ❑ Tak  ❑ Nie  ❑ Nie wiem |
| Jeśli TAK – Jaka była relacja tej osoby z Twoim dzieckiem?__________________________________  Ile lat miało Twoje dziecko? Po raz pierwszy ________ Ostatni raz ________ Najbardziej stresujący ________  Czy Twoje dziecko było pod silnym wpływem co najmniej jednego z tych zdarzeń? ❑ Tak ❑ Nie ❑ Nie wiem |  |
| 2.1 Czy kiedykolwiek ktoś napastował fizycznie Twoje dziecko, np. bił, popychał, dusił, potrząsał, gryzł, przypalał? Lub karał fizycznie Twoje dziecko i spowodował rany fizyczne lub siniaki. Lub atakował Twoje dziecko z bronią palną, nożem lub inną bronią? (Mógł to zrobić ktoś z rodziny lub z spoza rodziny). | ❑ Tak  ❑ Nie  ❑ Nie wiem |
| Jeśli TAK – Jaka była relacja tej osoby z Twoim dzieckiem?__________________________________  Czy użyto broni? ❑ Tak ❑ Nie ❑ Nie wiem  Ile lat miało Twoje dziecko? Po raz pierwszy ________ Ostatni raz ________ Najbardziej stresujący ________  Czy Twoje dziecko było pod silnym wpływem co najmniej jednego z tych zdarzeń? ❑ Tak ❑ Nie ❑ Nie wiem |  |
| 2.2 Czy kiedykolwiek ktoś bezpośrednio groził Twojemu dziecku, że zrobi mu poważną fizyczną krzywdę? | ❑ Tak  ❑ Nie  ❑ Nie wiem |
| Jeśli TAK – Jaka była relacja tej osoby z Twoim dzieckiem?__________________________________  Czy grożono z użyciem broni? ❑ Tak ❑ Nie ❑ Nie wiem  Ile lat miało Twoje dziecko? Po raz pierwszy ________ Ostatni raz ________ Najbardziej stresujący ________  Czy Twoje dziecko było pod silnym wpływem co najmniej jednego z tych zdarzeń? ❑ Tak ❑ Nie ❑ Nie wiem |  |
| 2.3 Czy Twoje dziecko zostało kiedykolwiek napadnięte lub próbowano je okraść? Lub czy było obecne kiedy członek rodziny lub inny opiekun lub przyjaciel został napadnięty? | ❑ Tak  ❑ Nie  ❑ Nie wiem |
| Jeśli TAK – Kogo napadnięto? (Jeśli to nie było Twoje dziecko) ______________________________________________  Czy grożono z użyciem broni? ❑ Tak ❑ Nie ❑ Nie wiem  Ile lat miało Twoje dziecko? Po raz pierwszy ________ Ostatni raz ________ Najbardziej stresujący ________  Czy Twoje dziecko było pod silnym wpływem co najmniej jednego z tych zdarzeń? ❑ Tak ❑ Nie ❑ Nie wiem |  |
| 2.4 Czy kiedykolwiek ktoś uprowadził Twoje dziecko (włączając rodzica i krewnych)? Lub czy kiedykolwiek uprowadzony został ktoś bliski dziecku? | ❑ Tak  ❑ Nie  ❑ Nie wiem |
| Jeśli TAK – Kto został uprowadzony? (Jeśli to nie było Twoje dziecko) ______________________________________________  Jaka była relacja porywacza z Twoim dzieckiem?__________________________________  Ile lat miało Twoje dziecko? Po raz pierwszy ________ Ostatni raz ________ Najbardziej stresujący ________  Czy Twoje dziecko było pod silnym wpływem co najmniej jednego z tych zdarzeń? ❑ Tak ❑ Nie ❑ Nie wiem |  |
| 2.5 Czy Twoje dziecko kiedykolwiek zostało zaatakowane przez psa lub inne zwierzę? | ❑ Tak  ❑ Nie  ❑ Nie wiem |
| Jeśli TAK – Ile lat miało Twoje dziecko? Po raz pierwszy ________ Ostatni raz ________ Najbardziej stresujący ________  Czy w wyniku ataku dziecko zostało poważnie ranne? ❑ Tak ❑ Nie ❑ Nie wiem  Czy Twoje dziecko było pod silnym wpływem co najmniej jednego z tych zdarzeń? ❑ Tak ❑ Nie ❑ Nie wiem |  |
| 3.1 Czy Twoje dziecko kiedykolwiek widziało, słyszało, lub słyszało o osobach **w Twojej rodzinie,** które ze sobą walczyły, biły się, policzkowały, kopały lub popychały nawzajem. Lub strzelały do siebie z broni palnej, albo stosowały inny rodzaj niebezpiecznej broni? | ❑ Tak  ❑ Nie  ❑ Nie wiem |
| Jeśli TAK – Jaka była relacja tych osób z Twoim dzieckiem?_______________________________________________________________  Czy użyto broni? ❑ Tak ❑ Nie ❑ Nie wiem  Ile lat miało Twoje dziecko? Po raz pierwszy ________ Ostatni raz ________ Najbardziej stresujący ________  Czy Twoje dziecko widziało co się stało? ❑ Tak ❑ Nie ❑ Nie wiem  Czy Twoje dziecko było pod silnym wpływem co najmniej jednego z tych zdarzeń? ❑ Tak ❑ Nie ❑ Nie wiem |  |
| 3.2 Czy Twoje dziecko widziało lub słyszało osoby **w Twojej rodzinie** grożące sobie wyrządzeniem poważnej krzywdy? | ❑ Tak  ❑ Nie  ❑ Nie wiem |
| Jeśli TAK – Jaka była relacja tych osób z Twoim dzieckiem?__________________________________  Czy grożono z użyciem broni? ❑ Tak ❑ Nie ❑ Nie wiem  Ile lat miało Twoje dziecko? Po raz pierwszy ________ Ostatni raz ________ Najbardziej stresujący ________  Czy Twoje dziecko było obecne gdy padały groźby? ❑ Tak ❑ Nie ❑ Nie wiem  Czy Twoje dziecko było pod silnym wpływem co najmniej jednego z tych zdarzeń? ❑ Tak ❑ Nie ❑ Nie wiem |  |
| 3.3 Czy Twoje dziecko kiedykolwiek dowiedziało się jak członek rodziny został aresztowany, uwięziony, lub zabrany (przez policję, żołnierzy lub inne służby)? | ❑ Tak  ❑ Nie  ❑ Nie wiem |
| Jeśli TAK – Jaka była relacja tych osób z Twoim dzieckiem?__________________________________  Ile lat miało Twoje dziecko? Po raz pierwszy ________ Ostatni raz ________ Najbardziej stresujący ________  Czy Twoje dziecko było obecne gdy padały groźby? ❑ Tak ❑ Nie ❑ Nie wiem  Czy Twoje dziecko było obecne gdy przyszła policja? ❑ Tak ❑ Nie ❑ Nie wiem  Czy Twoje dziecko było pod silnym wpływem co najmniej jednego z tych zdarzeń? ❑ Tak ❑ Nie ❑ Nie wiem |  |
| 4.1 Czy Twoje dziecko kiedykolwiek widziało lub słyszało, jak **osoby spoza rodziny** walczą, biją się, popychają, lub atakują siebie nawzajem? Lub widziało lub słyszało o przemocy jak bójki, strzelaniny, lub napady, które miały miejsce w otoczeniu ważnym dla Twojego dziecka, jak szkoła, Twoje sąsiedztwo, lub sąsiedztwo kogoś ważnego dla Twojego dziecka? | ❑ Tak  ❑ Nie  ❑ Nie wiem |
| Jeśli TAK – Jaka była relacja tych osób z Twoim dzieckiem?__________________________________  Ile lat miało Twoje dziecko? Po raz pierwszy ________ Ostatni raz ________ Najbardziej stresujący ________  Czy Twoje dziecko widziało, co się stało? ❑ Tak ❑ Nie ❑ Nie wiem Gdzie to się stało?______________________________  Czy Twoje dziecko było pod silnym wpływem co najmniej jednego z tych zdarzeń? ❑ Tak ❑ Nie ❑ Nie wiem |  |
| 4.2 Czy Twoje dziecko było kiedykolwiek bezpośrednio narażone na wojnę, konflikt zbrojny lub terroryzm? | ❑ Tak  ❑ Nie  ❑ Nie wiem |
| Jeśli TAK – Ile lat miało Twoje dziecko? Po raz pierwszy ________ Ostatni raz ________ Najbardziej stresujący ________  Czy Twoje dziecko było pod silnym wpływem co najmniej jednego z tych zdarzeń? ❑ Tak ❑ Nie ❑ Nie wiem |  |
| 4.3 Czy Twoje dziecko kiedykolwiek widziało lub słyszało o wojnie lub aktach terroryzmu w telewizji lub w radiu? | ❑ Tak  ❑ Nie  ❑ Nie wiem |
| Jeśli TAK – Ile lat miało Twoje dziecko? Po raz pierwszy ________ Ostatni raz ________ Najbardziej stresujący ________  Czy Twoje dziecko było pod silnym wpływem co najmniej jednego z tych zdarzeń? ❑ Tak ❑ Nie ❑ Nie wiem |  |
| 5.1 Czy kiedykolwiek ktoś **zmuszał** Twoje dziecko do oglądania lub robienia czegoś związanego z seksem (jak dotykanie w seksualny sposób, obnażanie się lub masturbowanie przy dziecku, udział w stosunku płciowym) | ❑ Tak  ❑ Nie  ❑ Nie wiem |
| Jeśli TAK – Jaka była relacja tych osób z Twoim dzieckiem?__________________________________  Czy stosowano przemoc fizyczną? ❑ Tak ❑ Nie ❑ Nie wiem Czy użyto broni? ❑ Tak ❑ Nie ❑ Nie wiem  Ile lat miało Twoje dziecko? Po raz pierwszy ________ Ostatni raz ________ Najbardziej stresujący ________  Czy Twoje dziecko było pod silnym wpływem co najmniej jednego z tych zdarzeń? ❑ Tak ❑ Nie ❑ Nie wiem |  |
| 5.2 Czy Twoje dziecko kiedykolwiek było obecne, gdy ktoś był zmuszany do jakiejkolwiek aktywności seksualnej? | ❑ Tak  ❑ Nie  ❑ Nie wiem |
| Jeśli TAK – Jaka była relacja tych osób z Twoim dzieckiem? Ofiara: __________________________________ Napastnik: _________________________________  Czy stosowano przemoc fizyczną? ❑ Tak ❑ Nie ❑ Nie wiem Czy użyto broni? ❑ Tak ❑ Nie ❑ Nie wiem  Ile lat miało Twoje dziecko? Po raz pierwszy ________ Ostatni raz ________ Najbardziej stresujący ________  Czy Twoje dziecko było pod silnym wpływem co najmniej jednego z tych zdarzeń? ❑ Tak ❑ Nie ❑ Nie wiem |  |
|  |  |
| 6.1 Czy Twojemu dziecko kiedykolwiek wielokrotnie powtarzano, że jest niedobre, krzyczano na nie w przerażający sposób, lub ktoś mu groził, że je porzuci, odejdzie lub odeśle je daleko? | ❑ Tak  ❑ Nie  ❑ Nie wiem |
| Jeśli TAK – Jaka była relacja osoby z Twoim dzieckiem ________________________________________________________  Ile lat miało Twoje dziecko? Po raz pierwszy ________ Ostatni raz ________ Najbardziej stresujący ________  Czy Twoje dziecko było pod silnym wpływem co najmniej jednego z tych zdarzeń? ❑ Tak ❑ Nie ❑ Nie wiem |  |
| 6.2 Czy Twoje dziecko kiedykolwiek przeżyło okres, w którym brakowało mu właściwej opieki (np. nie miało wystarczająco dużo jedzenia lub picia, brakowało schronienia, zostało zostawione samo gdy było za małe, żeby zatroszczyć się o siebie, lub było pozostawione z opiekunem nadużywającym narkotyków lub alkoholu) | ❑ Tak  ❑ Nie  ❑ Nie wiem |
| Jeśli TAK – Ile lat miało Twoje dziecko? Po raz pierwszy ________ Ostatni raz ________ Najbardziej stresujący ________  Czy Twoje dziecko było pod silnym wpływem co najmniej jednego z tych zdarzeń? ❑ Tak ❑ Nie ❑ Nie wiem |  |
| 7.1 Czy zdarzyły się Twojemu dziecku jakieś inne stresujące rzeczy? | ❑ Tak  ❑ Nie  ❑ Nie wiem |
| Jeśli TAK – Opisz krótko te rzeczy:___________________________________________________________________________________________________________ ______________________________________________________________________________________________________________________________________  Ile lat miało Twoje dziecko? Po raz pierwszy ________ Ostatni raz ________ Najbardziej stresujący ________  Czy Twoje dziecko było pod silnym wpływem co najmniej jednego z tych zdarzeń? ❑ Tak ❑ Nie ❑ Nie wiem |  |

Tłumaczenie (wersja eksperymentalna):

Tomasz Hanć

Zakład Biologii Rozwoju Człowieka, Wydział Biologii UAM, [tomekh@amu.edu.pl](mailto:tomekh@amu.edu.pl)
